# Targeting hepatic sulfane sulfur/hydrogen sulfide signaling pathway with α-lipoic acid to prevent diabetes-induced liver injury via upregulating hepatic CSE/3-MST expression

**DOI:** 10.1186/s13098-022-00921-x

**Published:** 2022-10-13

**Authors:** George J. Dugbartey, Karl K. Alornyo, Ismaila Adams, Stephen Atule, Richard Obeng-Kyeremeh, Daniel Amoah, Samuel Adjei

**Affiliations:** 1 Department of Pharmacology and Toxicology, School of Pharmacy, College of Health Sciences, University of Ghana, Legon, Accra, Ghana; 2 Department of Medical Pharmacology, University of Ghana Medical School, Korle-Bu, Accra, Ghana; 3 Department of Animal Experimentation, College of Health Sciences, Noguchi Memorial Institute for Medical Research, University of Ghana, Legon, Accra, Ghana

**Keywords:** Alpha-lipoic acid (ALA), Type 2 diabetes mellitus (T2DM), Diabetes-induced liver injury, Non-alcoholic fatty liver disease (NAFLD), Non-alcoholic steatohepatitis (NASH), Sulfane sulfur, H_2_S pathway

## Abstract

**Background:**

Diabetes-induced liver injury is a complication of diabetes mellitus of which there are no approved drugs for effective treatment or prevention. This study investigates possible hepatoprotective effect of alpha-lipoic acid (ALA), and sulfane sulfur/hydrogen sulfide pathway as a novel protective mechanism in a rat model of type 2 diabetes-induced liver injury.

**Methods:**

Thirty Sprague–Dawley rats underwent fasting for 12 h after which fasting blood glucose was measured and rats were randomly assigned to diabetic and non-diabetic groups. Type 2 diabetes mellitus (T2DM) was induced in diabetic group by administration of nicotinamide (110 mg/kg) and streptozotocin (55 mg/kg). Diabetic rats were treated daily with ALA (60 mg/kg/day p.o.) or 40 mg/kg/day DL-propargylglycine (PPG, an inhibitor of endogenous hydrogen sulfide production) for 6 weeks and then sacrificed. Liver, pancreas and blood samples were collected for analysis. Untreated T2DM rats received distilled water.

**Results:**

Hypoinsulinemia, hyperglycemia, hepatomegaly and reduced hepatic glycogen content were observed in untreated T2DM rats compared to healthy control group (p < 0.001). Also, the pancreas of untreated T2DM rats showed severely damaged pancreatic islets while liver damage was characterized by markedly increased hepatocellular vacuolation, sinusoidal enlargement, abnormal intrahepatic lipid accumulation, severe transaminitis, hyperbilirubinemia, and impaired hepatic antioxidant status and inflammation compared to healthy control rats (p < 0.01). While pharmacological inhibition of hepatic sulfane sulfur/hydrogen sulfide with PPG administration aggravated these pathological changes (p < 0.05), ALA strongly prevented these changes. ALA also significantly increased hepatic expression of hydrogen sulfide-producing enzymes (cystathionine γ-lyase and 3-mecaptopyruvate sulfurtransferase) as well as hepatic sulfane sulfur and hydrogen sulfide levels compared to all groups (p < 0.01).

**Conclusions:**

To the best of our knowledge, this is the first experimental evidence showing that ALA prevents diabetes-induced liver injury by activating hepatic sulfane sulfur/hydrogen sulfide pathway via upregulation of hepatic cystathionine γ-lyase and 3-mecaptopyruvate sulfurtransferase expressions. Therefore, ALA could serve as a novel pharmacological agent for the treatment and prevention of diabetes-induced liver injury, with hepatic sulfane sulfur/hydrogen sulfide as a novel therapeutic target.

## Background

Diabetes-induced liver injury is a common complication of diabetes mellitus due to impaired metabolism of glucose and lipids. As the liver is the hub for the metabolism of these essential biomolecules, intrahepatic alterations in their metabolism either dependent or independent of extrahepatic conditions lead to their abnormal accumulation in the liver [1]. Both type 1 (T1DM) and type 2 (T2DM) diabetic patients are at high risk of having abnormal deposition of free fatty acids (FFA) and triglycerides in the liver parenchyma, as well as elevated liver enzymes, hepatic fibrosis and an increase in morbidity and mortality due to diabetes-induced liver injury [2–5]. Also, non-alcoholic fatty liver disease (NAFLD; characterized by abnormal accumulation of lipids in liver cells) and its progressive form, non-alcoholic steatohepatitis (NASH), are common causes of hepatic dysfunction particularly in T2DM patients, even if they drink little or no alcohol. If left untreated or uncontrolled, NAFLD and NASH progress to cirrhosis (advanced scarring) and end-stage liver disease or hepatocellular carcinoma, hallmarked by hepatomegaly as one of the most common physical examination findings [6, 7]. Such hepatic damage is similar to that caused by heavy alcohol use. Early manifestations of NAFLD and NASH in diabetic patients include glycogenated nuclei usually in periportal hepatocytes, as well as steatosis, and the presence of immune cells such as lymphocytes and neutrophils in the liver [8].

There are studies showing that oxidative stress due to over-production of reactive oxygen species (ROS), and activation of inflammatory pathways play central roles in the development and progression of NAFLD and NASH [9, 10]. Oxidative damage to hepatocytes due to hepatic glucotoxicity activates a cascade of inflammatory events including the recruitment of immune cells in response to acute hepatic injury. ROS-mediated damage to subcellular structures such as the mitochondria causes impaired β-oxidation of FFA in the liver, leading to its accumulation, a condition known as steatosis [9, 10]. In addition, there is an increase in apoptotic hepatocytes and caspase-3 activation in the progression of NAFLD and NASH as a result of the cumulative effect of ROS-mediated damage and inflammation [11–13]. Despite all these intensive studies, there are currently no approved pharmacological agents for effective treatment of patients with NAFLD and NASH including diabetic patients. Besides, existing anti-diabetic and anti-hyperlipidemic drugs only retard the disease progression but do not prevent or reverse the pathology. Moreover, some of these conventional drugs have adverse effects ranging from bladder cancer, hepatotoxicity to lactic acidosis [14–16]. Therefore, identification of novel drugs or development new pharmacotherapeutic regimens aimed at effective treatment or prevention of the pathology with less severe adverse effects is “a holy grail” in diabetology.

Burgeoning evidence from human and animal studies including ours shows that alpha-lipoic acid (ALA), an organosulfur antioxidant synthesized from cysteine (an endogenous source of hydrogen sulfide) [17, 18], improves other diabetic complications when used alone or in combination with other agents [19–22]. In humans and animals, ALA is synthesized in the mitochondria of hepatocytes and other cell types, where it helps mitochondrial enzymes to convert nutrients including glucose and fatty acids into energy, thus functioning as a cofactor for mitochondrial enzymes in cellular bioenergetics [23]. As the liver is the main organ in energy homeostasis by converting nutrients absorbed by the intestine into glucose, fatty acids and other substrates, and storage of glycogen and lipids for the body’s use, it suggests that ALA plays a significant part in the homeostatic function of the liver in the metabolism of these biomolecules. ALA is also administered exogenously after which it is absorbed and converted enzymatically to dihydrolipoic acid (DHLA) [24, 25]. Results from two recent preclinical studies have shown that administration of ALA produces DHLA, which releases hydrogen sulfide (H_2_S) from sulfane sulfur (a reactive and labile divalent sulfur atom covalently bonded to another sulfur atom) in the homogenate of rat liver tissue [26, 27]. H_2_S, a gas recognized by its characteristic obnoxious odour of rotten eggs, is the third established member of gasotransmitter family next to nitric oxide and carbon monoxide, with biological usefulness and therapeutic potential at low nanomolar to middle micromolar concentrations [28, 29]. In the liver, H_2_S is principally synthesized by cystathionine γ-lyase (CSE) and 3-mecaptopyruvate sulfurtransferase (3-MST), a cytosolic and mitochondrial enzyme respectively, and their inhibition leads to various forms of chronic liver disease [30, 31]. Interestingly, these enzymes have been recently reported to also produce sulfane sulfur, suggesting that inhibitors of endogenous H_2_S production may also inhibit cellular sulfane sulfur levels. Just like ALA, H_2_S is also administered exogenously using H_2_S-containing compounds [32–34].

While there is a substantial body of evidence on the therapeutic benefits of H_2_S in various pathologies including diabetic conditions [35–37], it is unknown whether a relationship exists between H_2_S and ALA in diabetic liver disease. In this present study, we sought to investigate for the first time the effect of ALA on hepatic sulfane sulfur/H_2_S signaling pathway in a rat model of type 2 diabetes-induced liver injury.

## Methods

### Ethical statement

All the animal work in this study was conducted according to relevant national and international guidelines and was approved by the University of Ghana Institutional Animal Care and Use Committee (Protocol ID: UG-IACUC 002/19-20), adhering to protocol and maintaining quality assurance in accordance with good laboratory practice and ARRIVE guidelines (Animal Research: Reporting of In Vivo Experiments).

### Experimental animals

Thirty male Sprague–Dawley rats (Hsd:SD stock) weighing 180–220 g were purchased at 6–8 weeks of age from Noguchi Memorial Institute for Medical Research, University of Ghana, Legon, Accra, Ghana. The animals were housed in stainless steel cages (34 cm × 47 cm × 18 cm) with softwood shavings as bedding, under controlled ambient temperature (23 ± 2 °C), relative humidity (50 ± 10%) and light/dark condition (L:D cycle 12:12 h) in the Department of Animal Experimentation of the same Institute with ad libitum access to standard rat chow (Agricare Ltd, Kumasi, Ashanti Region, Ghana) and tap water.

### Experimental grouping and procedures

**Fig. 1:**
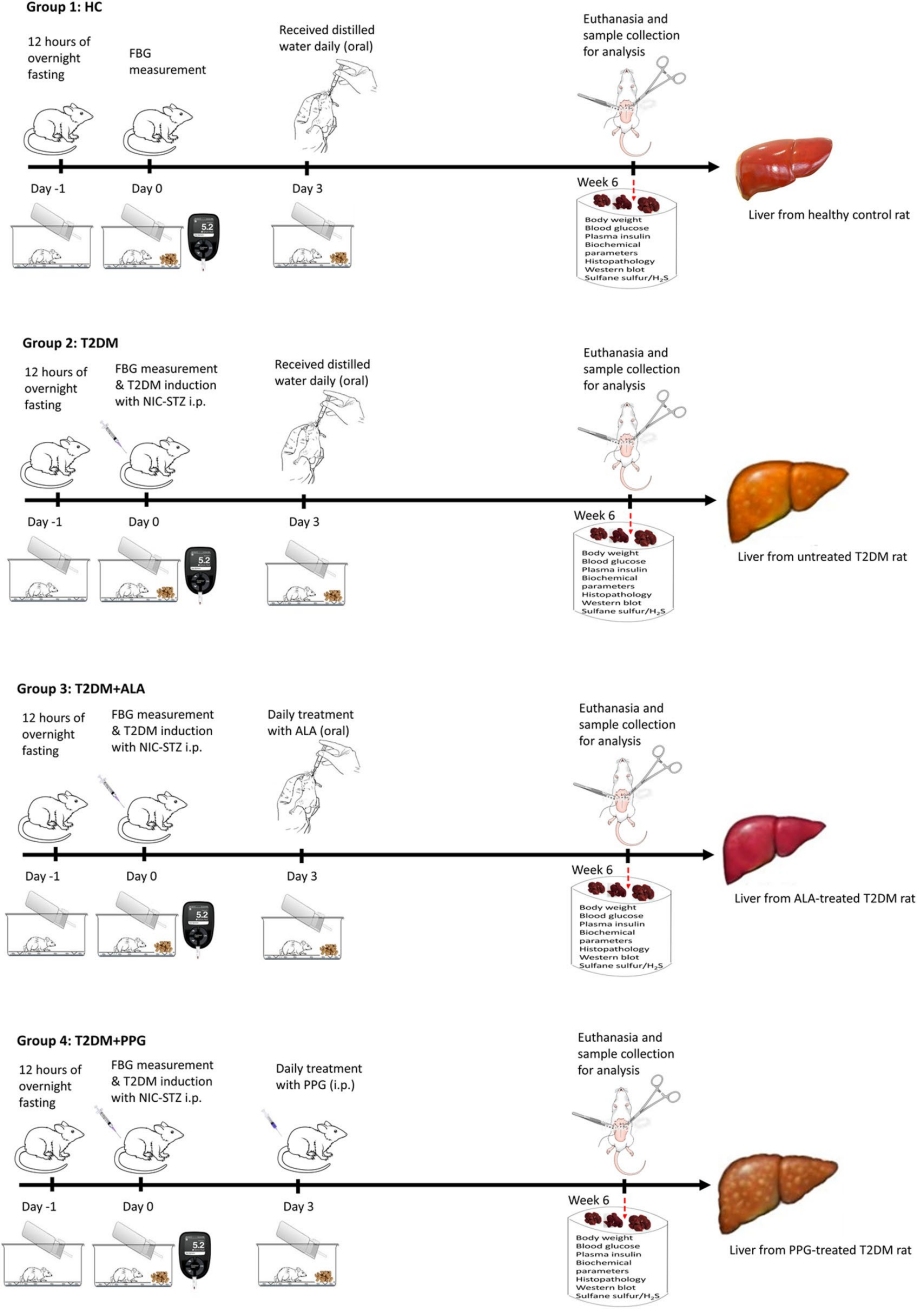
The rat model of type 2 diabetes mellitus (T2DM)-induced liver injury. Following 12 h of fasting, fasting blood glucose (FBG) was measured and T2DM was induced by nicotinamide (NIC; 110 mg/kg) and streptozotocin (STZ; 55 mg/kg) administration. T2DM was confirmed on day 3 after its induction. Diabetic rats received either oral administration of alpha-lipoic acid (T2DM + ALA; 60 mg/kg/day) or intraperitoneal injection of DL-propargylglycine (T2DM + PPG; 40 mg/kg/day) for 6 weeks. Untreated diabetic and non-diabetic rats received distilled water orally and served as diabetic (T2DM) and healthy control (HC) groups respectively. The rats were sacrificed after 6 weeks and samples were collected for analysis

The experimental procedure is illustrated in Fig. 1. In brief, rats underwent fasting for 12 h prior to induction of type 2 diabetes mellitus (T2DM), and blood samples were obtained by tail snipping for measurement of fasting blood glucose (FBG) with a portable hand-held glucometer (One Touch Select Plus^®^; LifeScan Inc., Zug, Switzerland). Seven of the rats were used as non-diabetic healthy control (HC; received distilled water orally) while T2DM was induced in the remaining 23 rats by intraperitoneal (*i.p.*) administration of 55 mg/kg streptozotocin (Sigma Aldrich, Missouri, USA) after i.p. injection of 110 mg/kg nicotinamide (Nanjing Yasnt Bio-Tech Co. Ltd—Nanjing, China). Only rats with FBG above 13.9 mmol/L (250 mg/dL) were considered as diabetic [38]. Following this, the diabetic rats were randomly divided into 3 groups being T2DM (untreated diabetic control [n = 9]; received distilled water), T2DM + ALA (received 60 mg/kg/day ALA by oral gavage; n = 7) and T2DM + PPG (received 40 mg/kg/day *i.p*; n = 7). Alpha-lipoic acid (ALA) was purchased from Nanjing Yasnt Bio-Tech Co. Ltd—Nanjing, China in powdered form, while DL-propargylglycine (PPG; an inhibitor of endogenous H_2_S production) from Sigma Aldrich Co, St. Louis, MO, USA. Clinical parameters such as body weight (BW) and food intake were measured every week in all groups till the day of sacrifice. All rats received their respective treatments (distilled water, ALA and PPG) daily for 6 weeks after which they were sacrificed under ketamine (60 mg/kg)/xylazine (10 mg/kg) anesthesia (*i.p*.) and blood samples were collected by cardiac puncture into EDTA tubes. The liver was harvested and weighed and relative liver weight [liver weight (LW)/BW ratio (%)] was computed. A piece of the liver tissue from each rat was snap-frozen and stored in a −80 °C freezer for western blot analysis, biochemical assays and measurement of sulfane sulfur and H_2_S production while another piece and pancreas were fixed in 10% neutral-buffered formalin for histological analysis. Glycosylated hemoglobin (HbA1c; a reliable marker and important index of average glycemia under diabetic conditions) was measured from whole blood using an automatic biochemical analyzer (Nycocard Reader, Axis Shield, Oslo, Norway).

### Plasma preparation and measurement of biochemical parameters

Whole blood sample was spun in a centrifuge at 3000 rpm for 15 min at 4 °C to obtain plasma. Upon obtaining plasma samples from the centrifugation step, plasma triglycerides, total cholesterol, low-density lipoproteins-cholesterol (LDL) and high-density lipoproteins-cholesterol (HDL) were measured with Mindray BS-200 Biochemistry Auto-analyzer, Shenzhen, China, while plasma insulin levels were measured using a radioimmunoassay (RIA) kit from Atom High-Tech Co., Ltd., Beijing, China, following the manufacturer's instructions. Plasma content of non-esterified free fatty acids (FFA) was measured using a non-esterified FFA colorimetric kit (Elabscience, USA) on an i-Mark microplate reader. Plasma levels of liver function markers such as alanine aminotransferase (ALT), aspartate aminotransferase (AST), alkaline phosphatase (ALP) and total bilirubin were measured with a Biochem-Immuno Autoanalyzer (TBA-40FR, Toshiba, Tokyo, Japan) while interleukin-1β (IL-1β), IL-6 and tumor necrosis factor-alpha (TNF-α) in plasma and liver tissue were measured by ELISA with the help of a DuoSet kit and in accordance with the manufacturer’s guide (Quantikine, R&D Systems, Minneapolis, MN, USA). Pancreatic β-cell function was assessed by computing HOMA-β using the formula: 20 × Insulin (μ IU/mL) ÷ Glucose (mmol/L) − 3.5).

### Measurement of intrahepatic and plasma antioxidant status

#### Determination of intrahepatic and plasma malondialdehyde levels

Hepatic malondialdehyde (MDA; a by-product of lipid peroxidation and an indicator of ROS production) was measured using thiobarbituric acid reactive substances (TBARS) method following the manufacturer’s guide (Abcam, Toronto, ON, Canada). Concisely, about 50 mg of liver tissue was homogenized in 100 μL phosphate-buffered saline (PBS) containing butylated hydroxytoluene (Cell Biolabs, Netherlands) and centrifuged at 10,000 g for 5 min at 4 °C. About 50 mL of the supernatant was collected and 50 mL SDS-Lysis solution (Cell Biolabs) was then added to MDA standards and incubated for 5 min at room temperature. Following this step, 125 mL of TBA reagent (Cell Biolabs) was added and incubated for 60 min at 95 °C and then cooled to room temperature for 5 min after which it was centrifuged at 1000 g for 15 min at 4 °C. The supernatant was collected and 150 μL of 2-Butanol (Merck, Darmstadt, Germany) was added to it, mixed for 2 min followed by centrifugation at 10,000 g for 5 min at 4 °C. Lipid peroxidation was determined from 200 μL of the butanol (organic) fraction by measuring optical density at 532 nm and expressed as ng/mg of liver tissue. The method was repeated using 200 μL of plasma samples, and the lipid peroxidation was measured at the same optical density.

#### Determination of intrahepatic and plasma glutathione content and superoxide dismutase activity

The content of glutathione (GSH) in liver tissue was measured using a spectrophotometric method following instructions from the test kit from Promega (Madison, WI, USA). In a nutshell, about 50 mg of frozen-kept liver tissue was homogenized in 1 mL of ice-cold 0.5% potassium chloride and sonicated for 1 min. The homogenate was centrifuged at 3000 rpm for 10 min at 4 °C and the supernatant was collected and used together with the GSH standard provided in the test kits. Hepatic GSH was quantified by spectophotometrically with the help of a SpectraMax 2 plate reader. The procedure was repeated using 150 μL of plasma samples. Measurement of SOD activity in liver and plasma was also done using 50 mg of frozen-kept liver tissue and 300 μL of plasma sample according to a previously described method by Xu et al. [39] and following the instructions from the test kit (Nanjing Kaiji Bio, Nanjing, China).

### Extraction and measurement of intrahepatic triglyceride, cholesterol, FFA and glycogen contents

#### Triglyceride

Extraction of triglyceride and total cholesterol was done using the Folch method described by Qasem et al. [40]. Summarily, lipids were extracted in a 2:1 mixture of chloroform and methanol, with a total volume of 5 mL after 50 mg of liver tissue was weighed and homogenized. Next, the extract was then centrifuged at 2500 g for 15 min and the supernatant was collected and placed in a chamber filled with nitrogen to allow evaporation to dryness, leaving a residue. About 5 mL of isopropyl alcohol containing 10% Triton X was added to the residue and centrifuged at 10 000 g for 10 min. The supernatant was collected and used for the measurement of triglyceride and cholesterol in the liver tissue with the help of a commercially available test kit and following the manufacturer’s guide (E1013, Applygen Technologies Inc., Beijing, China).

#### Free fatty acid

Extraction and determination of free fatty acid (FFA) in the liver were done as previously described [41]. In brief, 200 mg of liver tissue was homogenized in a 1 mL of lysis buffer containing 2 mmol/L NaCl, 200 μL of 20 mmol/L EDTA, and 50 mmol/L sodium phosphate buffer with a pH of 7.4. Following the homogenization step, 100 μL of the supernatant was mixed with 100 μL of tert-butyl alcohol and 50 μL of Triton X-100/methyl alcohol mixture (1:1 vol/vol) to extract the FFA and then measured using a colorimetric assay kit (Sigma-Aldrich, Ontario, Canada).

#### Glycogen

Measurement of hepatic glycogen content was done using a commercially available colorimetric glycogen assay kit adhering to the manufacturer’s instructions (Abcam, Toronto, Ontario, Canada). Briefly, 10 mg of liver tissue was rapidly homogenized with 200 µL ddH_2_O for 10 min on ice and then boiled for another 10 min to inactivate enzymes. The homogenate was centrifuged at 18,000 rpm for 10 min and the supernatant was collected. The supernatant (30 μL) was added to a 96-well plate and the volume was brought to 50 µL with glycogen hydrolysis buffer provided in the kit. A volume of 2 µL of hydrolysis enzyme mix was added to standard and samples, and mixed well, followed by incubation at room temperature for 30 min. Next, 48 µL of reaction mix was added to the standard and samples, and incubated at room temperature for 30 min, and absorbance of the solution was determined at 450 nm with a microplate reader.

### Measurement of intrahepatic sulfane sulfur and H_2_S contents

#### Measurement of hepatic sulfane sulfur

Hepatic sulfane sulfur was measured using cold cyanolysis method with colorimetric detection as previously described [42]. This is a method that involves a nucleophilic attack on a sulfur-sulfur bond by cyanide. Briefly, following cold homogenization on ice using 50 mg of liver tissue, a solution containing 720 μL of distilled water, 80 μL of 1 mol/L NH_3_, and 100 μL of 0.5 mol/L KCN was added to 100 μL of the tissue homogenate and mixed thoroughly. Next, the mixture was incubated at room temperature for 45 min after which 20 μL of 38% formaldehyde solution and 200 μL of Goldstein reagent containing Fe^3+^ cation were added and centrifuged at 12,000 g for 10 min. The supernatant was collected and the absorbance was measured at a wavelength of 460 nm. The pool of sulfane sulfur was determined from a calibration curve with 1 mmol/L KSCN.

#### *Measurement of hepatic and plasma H*_*2*_*S production*

The content of H_2_S in liver tissue was measured spectrophotometrically as previously described [43]. In short, about 50 mg of liver tissue was homogenized in cold KHPO_4_ buffer (1:10). A volume of 200 μL of the homogenate was mixed with 200 μL of 2 mmol/L pyridoxal 5′-phosphate, 200 μL of 10 mM L-cysteine and 200 μL of 10% trichloroacetic acid. Next, the mixture was incubated at 37 °C for 30 min after which zinc acetate (1% w/v, 250 μL 1%) was added to trap H_2_S. Following this step, *N,N*-dimethyl-p-phenylenediamine sulfate (20 mmol/L, 133 μL) in 7.2 mol/L HCl and FeCl_3_ (30 mmol/L, 133 μL) in 1.2 mol/L HCl were added. The resulting solution was transferred to a 96-well plate and the absorbance of the solution was determined at 670 nm. The H_2_S production was calculated against a standard curve of NaHS (0.1–100 μmol/L). Plasma H_2_S was measured using a sulfide ion selective electrode (Lazar Research Laboratories, Inc., Los Angeles, CA, USA) and a sulfide antioxidant buffer as we previously described [44].

### Western blotting

The expressions of hepatic CSE and 3-MST proteins and β-actin (house-keeping protein) were determined by western blot technique. To be brief, hepatic expression of these proteins on the nitrocellulose membranes were detected using CSE (1:1000; Abnova, USA), 3-MST (1:1000; Santa Cruz, The Netherlands) and β-actin (1:5000; Abcam, Canada) primary antibodies following an overnight incubation at 4 °C. Next, the membranes were washed three times with a washing buffer (Tris-buffered saline [TBS] with 0.004% Tween) and incubated with their respective HRP-linked secondary antibodies (1:1000) in TBS + Tween-20 supplemented with 3% bovine serum albumin (w/v) at room temperature for 1 h. The blots were developed with SuperSignal West Dura Extended Duration Substrate (Thermo Scientific, USA) and protein bands were visualized with a Gene Genome. The intensities of the target protein bands were quantified using GeneTools analysis software (Westburg B.V., Leusden, The Netherlands) and normalized against the intensi*ty of β-actin.*

### Histopathology

#### Hematoxylin/Eosin stain

Formalin-fixed liver tissue samples were processed for histological examination as we previously described [45]. Concisely, the samples were dehydrated in alcohol series (70%, 80%, 90% and 100%) followed by further dehydration in xylene. Following the dehydration step, the samples were embedded in molten paraffin wax and then cut from a similar site of the tissue and in a similar plane at 4 μm thick. Next, samples were rehydrated in a decreasing series of alcohol and distilled water and then stained with hematoxylin/eosin (HE) to evaluate morphological changes.

#### Oil red O stain

To visualize and examine triglyceride and other lipid deposits in diabetic liver tissue, frozen sections of liver tissue from each group were stained using Oil red O staining protocol. In brief, samples were cut at 8 mm thickness and air-dried on glass slides. The sections were then fixed in formalin and washed under running tap water for 5 min after which they were rinsed in 60% isopropanol. Next, the liver samples were stained with freshly prepared Oil red O solution for 15 min and then rinsed again in 60% isopropanol. Following this step, the samples were dipped briefly in hematoxylin and then rinsed with distilled water. The samples were mounted in an aqueous mounting medium and examined under a light microscope (Leica DM1000, Leica Microsystems, Morrisville, NC, USA) at 100 × magnification by two experienced hepatic pathologists in a double-blinded fashion independently. Fatty degeneration was scored by following the classification system according to Toblli et al. [46]. Thus, 0 (absent), 1 (mild; 10% of microscopic field), 2 (moderate: 25% of microscopic field), 3 (severe; 50% of microscopic field) and 4 (very severe; > 50% of microscopic field).

#### Periodic acid-Schiff stain

Pancreas tissue samples were dewaxed and stained with periodic acid-Schiff (PAS) reagent and counterstained briefly with Meyer’s hematoxylin. Both liver and pancreas tissue sections were examined under a light microscope (Leica DM1000, Leica Microsystems, Morrisville, NC, USA) at 400 × magnifications and the liver sections were scored on the degree of hepatocellular vacuolation, sinusoidal enlargement and liver fatty degeneration in a double-blinded fashion independently by two experienced hepatic pathologists as previously described [47].

### Statistical analysis

All results are shown as mean ± standard error of the mean (SEM). One-way analysis of variance (ANOVA) with Tukey post-hoc comparison test was used to evaluate differences between groups. Statistical calculations were done using GraphPad Prism analysis software (Prism 8, GraphPad Software, Inc., San Diego, CA, USA). The criterion for statistical significance between groups was set at p < 0.05.

## Results

### Maintenance of body weight and preservation of glycemic and insulin levels and intrahepatic glycogen content by ALA

**Fig. 2:**
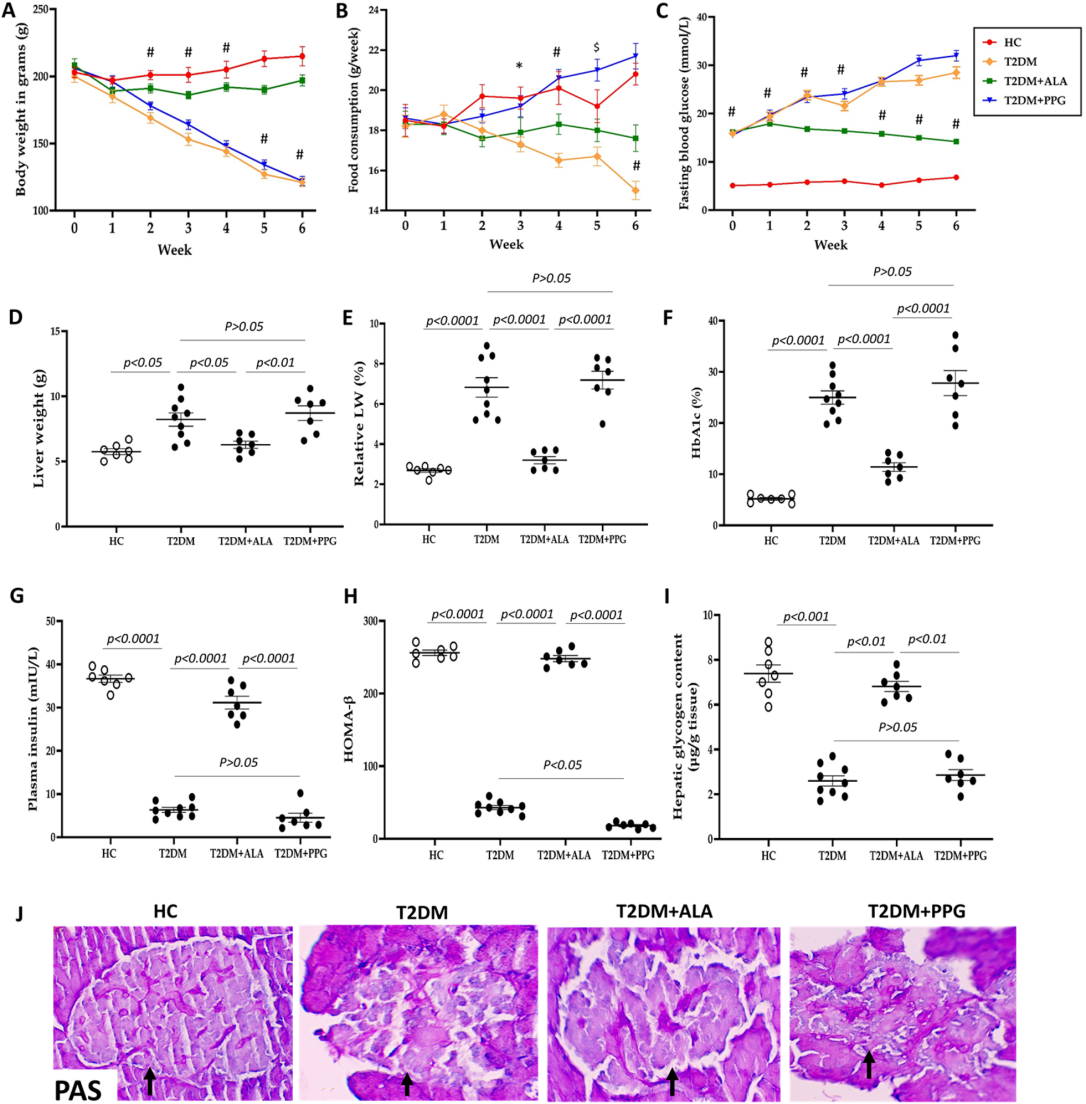
Body weight, glycemic status, plasma insulin and pancreas histology. **A** Body weight, **B** food consumption **C** fasting blood glucose **D** liver weight **E** relative liver weight, **F** HbA1c, **G** plasma insulin, **H** HOMA-β, **I** hepatic glycogen content and **J** representative micrographs of PAS-stained images of pancreatic tissues at 400 × magnification. Black arrows point to pancreatic islets. HC = Healthy control; T2DM = Untreated diabetic control; T2DM + ALA = Diabetic rats treated with alpha-lipoic acid; DT2M + PPG = Diabetic rats treated with propargylglycine. #p < 0.0001 vs. T2DM, $p < 0.001 vs. T2DM, *p < 0.05 vs. T2DM

We measured clinical parameters such as body weight, food intake, blood glucose, hepatic glycogen content, pancreatic β-cell function and plasma insulin in addition to pancreas histology under diabetic conditions and the effect of ALA. Induction of T2DM resulted in a progressive decline in body weight, food consumption and persistent hyperglycemia over the 6-week period, which correlated with marked increase in liver weight and relative liver weight compared to healthy control (HC) rats (Fig. 2A–F). In addition, untreated T2DM rats also showed hypoinsulinemia, significantly decreased β-cell function and hepatic glycogen content and severely damaged pancreatic islets in comparison with rats in the HC group (Fig. 2G–J;). Whereas administration of DL-propargylglycine (PPG) further aggravated these changes in T2DM + PPG rats although not significantly (Fig. 2A, D–G, I and J; p > 0.05) with the exception of HOMA-β, food intake and fasting blood glucose, which increased significantly over time (Fig. 2B, C and H), treatment with ALA in T2DM + ALA group of rats prevented these changes over the 6-week period, maintaining the levels of these parameters at HC levels (Fig. 2A–J; p > 0.05). Thus, ALA maintained body weight and preserved glycemic and insulin levels as well as hepatic glycogen content and pancreatic islet structure under diabetic conditions.

### Preservation of hepatic integrity by ALA

**Fig. 3:**
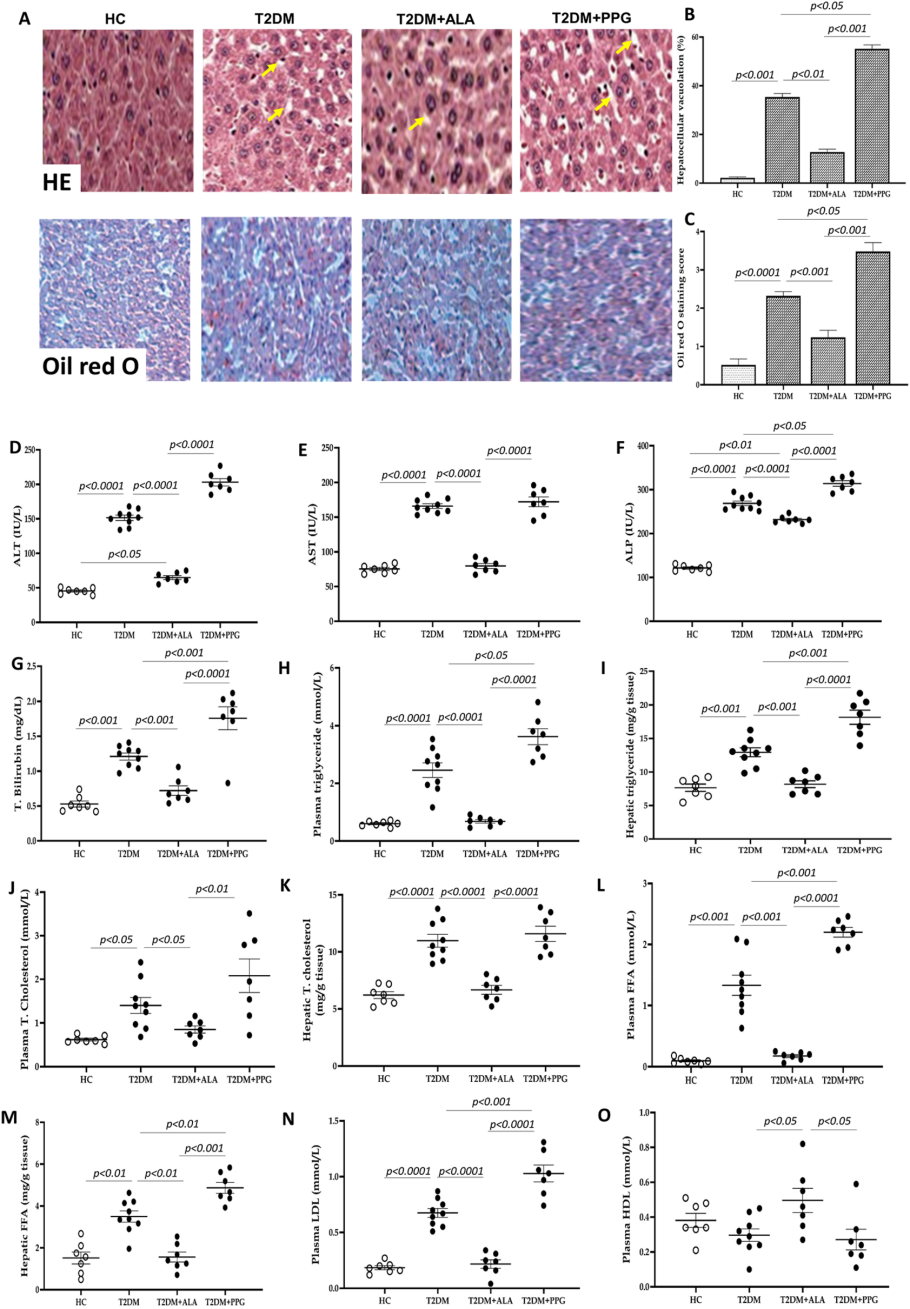
Liver histology, parameters of liver function and lipid profile **A** Representative photomicrographs of hematoxylin and eosin, and oil red O staining at 400 × and 100 × magnification respectively. Yellow arrows in HE images point to hepatocellular vacuolation and sinusoidal enlargement while red stains in oil red O images indicate triglyceride and other lipid accumulation. **B** and **C** Quantification of liver histology, **D** ALT, **E** AST, **F** ALP, **G** total bilirubin, **H** plasma triglyceride, **I** hepatic triglyceride, **I** plasma triglyceride, **J** plasma total cholesterol, **K** hepatic total cholesterol, **L** plasma FFA, **M** hepatic FFA, **N** plasma LDL and **O** plasma HDL. HC = Healthy control; T2DM = Untreated diabetic control; T2DM + ALA = Diabetic rats treated with alpha-lipoic acid; DT2M + PPG = Diabetic rats treated with propargylglycine

**Fig. 4:**
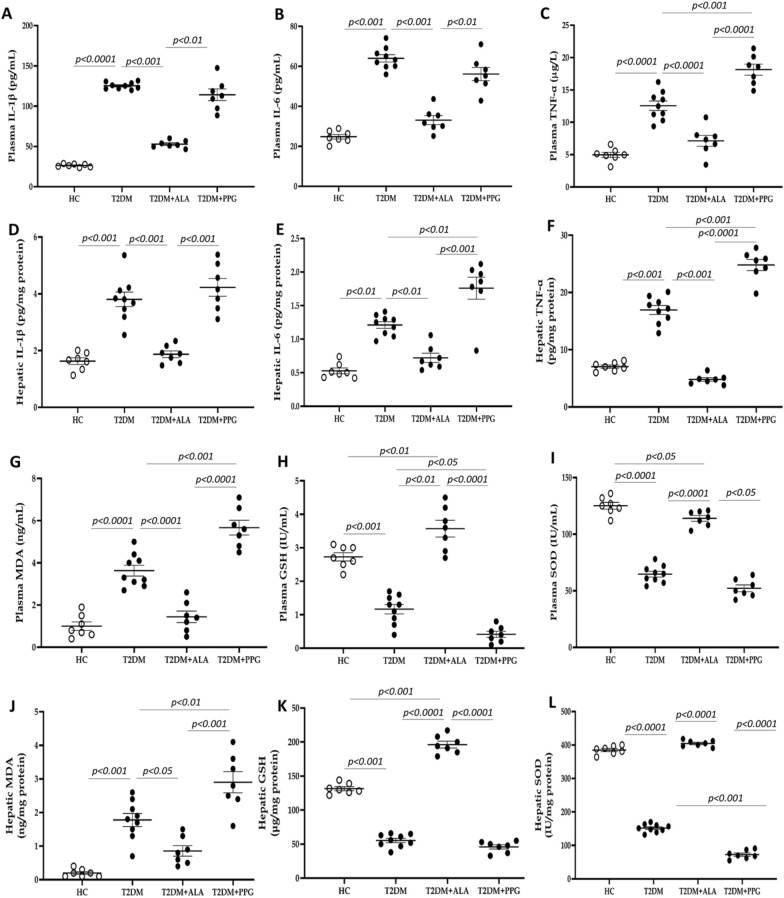
Inflammation and antioxidant status. **A** Plasma IL-1β **B** plasma IL-6, **C** plasma TNF-α, **D** hepatic IL-β, **E** hepatic IL-6, **F** hepatic TNF-α, **G** plasma MDA, **H** plasma GSH, **I** plasma SOD, **J** hepatic MDA, **K** hepatic GSH and **L** hepatic SOD. HC = Healthy control; T2DM = Untreated diabetic control; T2DM + ALA = Diabetic rats treated with alpha-lipoic acid; DT2M + PPG = Diabetic rats treated with propargylglycine

As abnormal liver morphology and function under uncontrolled diabetes are usually due to fatty changes in the diabetic liver, we investigated the effect of ALA on hepatic structure and function following induction of T2DM by performing liver histology, liver function test (ALT, AST, ALP, total bilirubin), and by determining hepatic lipid profile (triglyceride, total cholesterol, FFA, LDL and HDL), inflammation (IL-1β; IL-6 and TNF-α) and antioxidant status (MDA, GSH and SOD). 6 weeks of untreated T2DM resulted in severe liver injury evidenced by hepatocellular vacuolation, sinusoidal enlargement and liver fatty degeneration (Fig. 3A–C). This positively correlated with severe transaminitis, hyperbilirubinemia and impaired plasma and hepatic lipid profile characterized by hyperlipidemia and hepatic steatosis compared to HC rats (Fig. 3D–O). Consistent with these pathological changes are significantly increased levels of plasma and hepatic pro-inflammatory cytokines and impaired hepatic antioxidant status marked by increased levels of IL-1β, IL-6, TNF-α and MDA and decreased GSH content and SOD activity relative to rats in HC group (Fig. 4A–L). While these pathological changes became worse in PPG-treated diabetic rats (Figs. 3A–O, 4A–L), ALA treatment protected against diabetes-induced liver injury by strongly preserving hepatic integrity, which was not statistically significant compared to that in HC rats with the exception of ALP and hepatic GSH which were significantly higher in ALA-treated rats than HC rats (Fig. 3A–O, 4A–L). Taken together, administration of ALA preserved hepatic integrity under T2DM conditions.

### Activation of intrahepatic sulfane sulfur/H_2_S signaling pathway by ALA

**Fig. 5:**
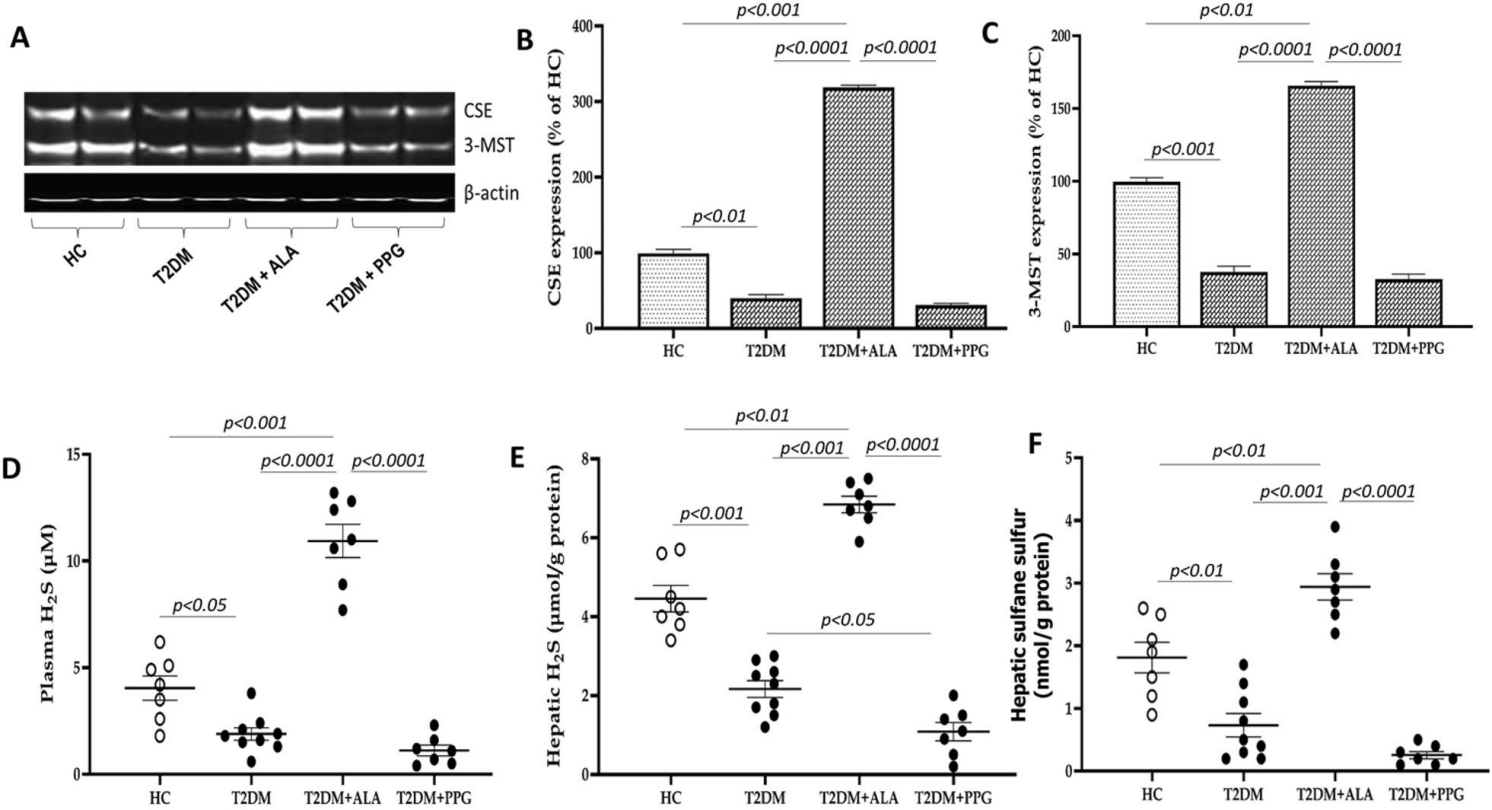
Western blot and hepatic sulfane sulfur/H_2_S system. **A** Image of a Western blot showing hepatic expression of CSE and 3-MST. Quantification of **B** CSE and **C** 3-MST. Measurement of **D** plasma H_2_S, **E** hepatic H_2_S and **F** hepatic sulfane sulfur. HC = Healthy control; T2DM = Untreated diabetic control; T2DM + ALA = Diabetic rats treated with alpha-lipoic acid; DT2M + PPG = Diabetic rats treated with propargylglycine

To investigate the potential involvement of hepatic sulfane sulfur/H_2_S as a mechanism in diabetes-induced liver injury and the effect of ALA, we measured these two sulfur species and H_2_S-producing enzymes (CSE and 3-MST) in the liver tissue. Untreated T2DM for 6 weeks significantly downregulated the expression of hepatic CSE and 3-MST enzymes, resulting in markedly reduced levels of hepatic sulfane sulfur and endogenous H_2_S in plasma and liver tissue in comparison with HC rats (Fig. 5A–F; p < 0.05). PPG administration further reduced plasma H_2_S and hepatic sulfane sulfur levels, with a significant reduction in hepatic H_2_S (Fig. 5D–F) while it also reduced hepatic CSE and 3-MST expression levels but not significantly (Fig. 5A–C; p > 0.05). However, treatment with ALA strongly upregulated the expression of these enzymes and markedly increased hepatic sulfane sulfur and H_2_S levels even above the levels in HC rats (Fig. 5A–F; p < 0.01). In summary, ALA administration activated hepatic sulfane sulfur/H_2_S signaling pathway, and thereby contributed to the prevention of diabetes-induced hepatic injury in ALA-treated rats.

## Discussion

Several lines of preclinical and clinical evidence have shown that the liver is one of the organs greatly affected by diabetes mellitus. However, there are currently no approved pharmacological agents for effective treatment or prevention of diabetes-induced liver injury. In this study, we report that 6 weeks of untreated type 2 diabetes mellitus (T2DM) in rats causes liver injury similar to the clinical version of non-alcoholic fatty liver disease (NAFLD) and non-alcoholic steatohepatitis (NASH; a progressive and aggressive form of NAFLD), both of which are characterized principally by hyperlipidemia and abnormal intrahepatic lipid accumulation without excessive alcohol intake. Hepatic histopathological alterations were evidenced by hepatocellular vacuolation, sinusoidal enlargement and liver fatty degeneration. Gross examination revealed hepatomegaly while functional changes were evidenced by severe transaminitis, hyperbilirubinemia, and impaired lipid profile. Other pathological changes include glycogen storage disruption, impaired hepatic antioxidant status and activation of pro-inflammatory pathway. We present evidence from a murine model of T2DM (one of the most common etiologies of NAFLD and NASH in humans) suggesting that suppression of hepatic sulfane sulfur/hydrogen sulfide (H_2_S) signaling pathway contributes to diabetes-induced liver injury, and that the activation of this pathway by alpha-lipoic acid (ALA) mediates hepatoprotection under diabetic condition. Importantly, pharmacological blockage of sulfane sulfur/H_2_S signaling pathway with DL-propargylglycine (PPG) resulted in further liver injury, with more lipid accumulation in the liver and plasma as seen in patients with NAFLD and NASH.

In the present study, treatment of T2DM rats with ALA for 6 weeks upregulated hepatic expression of the H_2_S-producing enzymes, CSE and 3-MST, and increased the levels of hepatic H_2_S and sulfane sulfur (the precursor of H_2_S), and thus contributed to hepatoprotection against diabetes-induced liver injury. Our observation is consistent with two previous studies in which administration of ALA increased sulfane sulfur and H_2_S levels in rat liver homogenates [26, 27]. Moreover, ALA treatment has been reported to restore bioenergetic effect of CSE and 3-MST and protected vascular smooth muscle cells in diabetic models similar to ours, in which hyperglycemia impaired CSE/H_2_S and 3-MST/H_2_S pathways [48, 49]. As observed in the present study, untreated T2DM as well as pharmacological inhibition of sulfane sulfur/H_2_S pathway strongly impaired hepatic H_2_S production, characterized by downregulation of hepatic CSE and 3-MST expressions and reduced levels of sulfane sulfur and endogenous H_2_S, and contributed to the development of NAFLD and NASH in diabetic rats, while ALA prevented this pathology. This finding corroborates those of several preclinical models of NAFLD and NASH in which hepatic CSE and 3-MST protein and gene expressions were markedly downregulated while activation of H_2_S pathway by H_2_S donor compounds protected the liver and prevented further progression of NAFLD and NASH [50–52]. Beyond the boundaries of these exciting preclinical findings, a placebo-controlled randomized clinical trial involving daily administration of ALA either alone or in combination with vitamin E for 6 months provided hepatoprotection in patients with NAFLD and NASH by reducing the levels of plasma triglyceride and tumor necrosis factor-alpha (TNF-α; a pro-inflammatory cytokine), and improved steatosis scores compared to placebo-control patients [19]. ALA also improved serum insulin levels in patients with NAFLD by reducing insulin resistance in two recent double-blind, placebo-controlled, randomized clinical trials [53, 54], and increased serum H_2_S levels, leading to improved glycemic status in T2DM patients [48]. In addition, ALA is currently under phase 4 clinical trial for the treatment of NAFLD (clinicaltrials.gov).

It is important to note that the hepatoprotective effect of ALA under diabetic conditions is linked to its therapeutic properties including anti-hyperglycemic, anti-apoptotic, anti-hyperlipidemic, antioxidant, anti-inflammatory and anti-fibrotic properties, which can be attributed to its ability to activate sulfane sulfur/H_2_S pathway. As shown in our study, impaired insulin secretion due to the destruction of cells in the pancreatic islet of Langerhans, particularly insulin-secreting pancreatic β-cells, resulted in hypoinsulinemia and persistent hyperglycemia in T2DM rats over the 6-week period, and further worsened following inhibition of sulfane sulfur/H_2_S pathway with PPG administration. However, ALA administration protected the β-cells and activated insulin secretion, thereby maintaining normoglycemia. As ALA activated sulfane sulfur/H_2_S pathway in our study, Takahashi et al. [55] observed that reactive sulfur species such as sulfane sulfur and H_2_S activate insulin secretory pathway via regulation of tRNA methylthiolation, and inhibited high glucose- and hyperglycemia-induced apoptosis of insulin-secreting pancreatic β-cells [56, 57]. These observations including ours, refute previous reports suggesting that H_2_S in pancreatic islets inhibits insulin secretion by activating adenosine triphosphate (ATP)-dependent potassium (K_ATP_) channels [58, 59]. Although H_2_S is known to activate and open K_ATP_ channels under various conditions, it is possible that these contradictory reports on the effect of H_2_S on insulin secretion could be due to the different doses of different H_2_S donor compounds used in these studies, thus resulting in different concentrations of total H_2_S in the insulin-secreting β-cells. This assertion is supported by the findings of Wu and colleagues [60], who attributed the inhibition of insulin secretion by H_2_S to its over-production. It is also possible that ALA has other mechanisms for increased insulin secretion besides its interaction with K_ATP_ channels such as increased phosphorylation of insulin receptor-β and insulin receptor substrate-1 as well as upregulation of glucose-stimulated insulin secretion (GSIS) genes glucokinase, glucose transporter 2 and Kir6.2 in the insulin signaling pathway, which have been reported to restore insulin secretion and protected islet cells under high glucose exposure [61]. Moreover, a phase 4 clinical trial is currently underway to determine whether ALA administration prevents or attenuates fatty acid-induced impairment of insulin secretion and insulin sensitivity (clinicaltrails.gov).

The anti-hyperglycemic effect of ALA as observed in our study, is also linked to its ability to activate insulin receptors and enhance their activity, hence ALA could be considered an insulin-mimetic agent. Moreover, ALA has been shown to be actively involved in glucose-regulating mechanisms by functioning as an insulin sensitizer in skeletal muscles [62], facilitating translocation of glucose transporter protein subtype 4 (GLUT 4) to the plasma [45] and activating adenosine monophosphate-activated protein kinase (AMPK) [62], a widely-recognized enzyme that regulates hepatic gluconeogenesis and cellular energy homeostasis by inhibiting gluconeogenic genes and enzymes which are abnormally regulated in gluconeogenesis pathway in T2DM [63]. In addition, as ALA activated sulfane sulfur/H_2_S system, H_2_S has been reported to attenuate hyperglycemia-induced vascular inflammation via AMPK activation [64], raising the possibility that ALA could serve a therapeutic purpose in other complications of chronic hyperglycemia of diabetes. Our result in the present study also includes increased intrahepatic glycogen content in ALA-treated T2DM rats, which was significantly reduced in untreated and PPG-treated T2DM rats, similar to findings from previous rat models of T2DM [65, 66]. This implies glycogen storage disruption in our T2DM model, which is commonly observed in diabetic patients with glycogen hepatopathy or liver cirrhosis (the advanced stage of NAFLD and NASH). It further suggests that ALA could maintain glucose homoeostasis under diabetic conditions by reducing gluconeogenesis and increasing glycogenesis, a pathway for disposal of excess glucose. Although our study did not include molecular mechanisms underlying this effect of ALA, inhibition of intrahepatic gluconeogenesis and increased glycogenesis have been associated with activation of AMPK signaling [67], a pathway that is also activated by ALA.

In addition to its anti-hyperglycemic effect, ALA also ameliorated hepatic steatosis and hypertriglyceridemia under diabetic conditions by stimulating triacylglycerol clearance and downregulating hepatic triacylglycerol secretion [68]. This aligns with our observation, in which ALA prevented the accumulation of triglyceride, cholesterol and free fatty acids (FFA) in the liver and plasma of T2DM rats. As ALA increased hepatic sulfane sulfur and endogenous H_2_S levels, with upregulated hepatic CSE and 3-MST expressions, thereby preventing hepatic lipid accumulation, similar observations have been made in previous studies. For example, it has been shown that downregulation of hepatic CSE and 3-MST and reduced endogenous H_2_S levels in mice and humans contribute to hypertriglyceridemia and accumulation of other lipids in the liver [69–71]. In the same studies, the authors reported that activation of the H_2_S pathway with H_2_S donor compounds including ALA reduced lipid accumulation in hepatocytes and improved NAFLD and NASH by stimulating hepatic autophagy through activation of AMPK signaling pathway dependent or independent of mammalian target of rapamycin (mTOR; a central regulator of cellular homeostasis) as well as via SIRT1/LKB1/AMPK pathway [69–71]. While these pathways were beyond the scope of our study, it can be inferred that activation of the hepatic sulfane sulfur/H_2_S pathway by ALA in the present study may have activated these molecular pathways and possibly other potential lipid-lowering pathways in the diabetic liver, thus reducing intrahepatic lipid content and improving NAFLD and NASH. The anti-hyperlipidemic effect of ALA is also associated with upregulation of short-, medium- and long-chain fatty acid metabolic processes (Acot1, Acot2, Acsf2, and Crat) and downregulation of lipogenic genes such as Pnpla3, Pnpla5, Elovl6, Acly and Gpam, which are major genetic risk factors for developing NAFLD [72]. These transcriptional changes may be partly responsible for the result in the present study, suggesting that inhibition of these genes by gene therapy or pharmacotherapy may be a novel approach for the treatment or prevention of NAFLD in diabetic patients as well as in patients with other metabolic diseases involving hyperlipidemia. Other mechanisms such as alterations in triglyceride secretion by liver cells due to malfunction of lipoproteins, and hyperglycemia-induced hepatic upregulation of GLUT2, which transports excess sugar from the blood into the liver and converted into excess fat, have been suggested as contributory factors in the development of NAFLD and NASH in diabetic patients [73]. Therefore, our results raise the possibility that ALA might have inhibited these mechanisms, leading to the improved lipid profile in our T2DM rats.

Besides the anti-hyperglycemic and anti-hyperlipidemic effects of ALA discussed above, we also observed in the present study that its hepatoprotective effect under diabetic conditions is partly due to its antioxidant and anti-inflammatory properties. Induction of T2DM in our model changed the intrahepatic oxidative balance over the 6-week period. This was hallmarked by markedly elevated levels of malondialdehyde (MDA; a by-product of lipid peroxidation and indicator of reactive oxygen species [ROS] production) and significantly reduced glutathione (GSH) content and superoxide dismutase (SOD) activity, thus overwhelming hepatic antioxidant defence system. The impaired intrahepatic antioxidant status correlated with pathological changes in liver morphology and function (increased ALT, AST and ALP, and total bilirubin) and inflammation (increased IL-1β, IL-6 and TNF-α levels). Our observations of the pathological changes in the diabetic liver in the present study are closely similar to those observed in chronic stress in liver cells. It is known that increased lipid peroxidation and free fatty acid oxidation lead to over-production of ROS-induced oxidative stress, which activates several stress-sensitive kinase signaling cascades such as JNK and IKKβ [74]. Also, ROS act on unsaturated fatty acids, and initiates lipid peroxidation, leading to changes in the fluidity and permeability of the liver cell membrane. This creates a suitable local environment for inflammatory infiltration, resulting in liver inflammation and activation of fibrotic and necrotic pathways that favor pathogenesis and progression of NAFLD and NASH [74].

As a potent antioxidant, which is often referred to as the “antioxidant of antioxidants” due to its ability to activate and recruit other antioxidants, ALA in the present study significantly improved hepatic antioxidant status and inflammation by decreasing the levels of ROS and pro-inflammatory cytokines, and increasing GSH and SOD levels, and thus contributing to hepatoprotection similar to previous reports in other liver disease models [75, 76]. Our result provides experimental support for the observations in two clinical trials in which antioxidant supplements significantly reduced transaminitis, hepatic fat accumulation and liver stiffness, and improved lipid profile in patients with NAFLD [77, 78]. It also confirms that induction of oxidative stress and activation of inflammatory cascade are crucial in the development and progression of NAFLD and NASH, and that activation of the H_2_S system indeed triggers hepatic antioxidant defence against NAFLD and NASH. Moreover, it has been shown that both ALA and other compounds containing H_2_S activate K_ATP_ channels, which in turn activate antioxidant and anti-inflammatory pathways, leading to organ protection [79–81]. It is also possible that the activation of antioxidant and anti-inflammatory pathways by ALA may be via other mechanisms besides its interaction with K_ATP_ channels such as activation of the transcription factor nuclear erythroid 2-related factor 2 (Nrf2; a regulator of transcription of antioxidants and other cytoprotective genes) as well as inhibition of nuclear factor-kappaB (NF-κB; a major protein complex that mediates inflammation by influencing pro-inflammatory cytokine production) and inhibiting the phosphorylating action of mitogen-activated protein kinase (MAPK; another major pro-inflammatory mediator) [82, 83]. Other studies also reported anti-fibrotic effect of ALA and H_2_S in animal models of hepatic fibrosis including diabetic animals by inhibiting the expression of the fibrotic proteins, transforming growth factor-beta1 (TGF-β1) and alpha-smooth muscle actin (α-SMA) [84, 85]. Although sulfane sulfur was not measured in these studies, it should be alluded that most of the studies that report increased levels of endogenous H_2_S production by H_2_S donor compounds also observe corresponding increases in cellular sulfane sulfur levels [86, 87]. Besides, sulfane sulfur and H_2_S always co-exist, and recent investigations suggest that some of the physiological processes previously reported to be regulated by H_2_S are actually mediated by sulfane sulfur [88, 89]. Moreover, our result also suggests that pharmacological inhibition of endogenous H_2_S also reduces sulfane sulfur levels. Taken together, the hepatoprotective effect of ALA in the present study may, at least in part, be due to its antioxidant, anti-inflammatory and anti-fibrotic properties.

While our results are exciting, we were unable to examine the diabetic liver at the subcellular level due to technical challenges. Such an assessment would have provided additional information on microvascular fatty degeneration as well as ultrastructural progressive impairment in the diabetic liver including mitochondria and other intracytoplasmic organelles of our T2DM rats.

## Conclusion

To sum up, we have demonstrated for the first time that ALA prevents diabetes-induced liver injury by activating hepatic sulfane sulfur/H_2_S pathway via upregulation of hepatic CSE and 3-MST protein expression. This constitutes a novel mechanism in the pharmacological treatment or prevention of NAFLD and NASH under diabetic conditions. Considering that there is significant hepatic involvement in diabetes mellitus coupled with the lack of approved pharmacological agents for diabetes-induced liver injury, our results provide experimental support for the idea that ALA or novel drugs containing sulfane sulfur and H_2_S with anti-hyperglycemic, anti-hyperlipidemic, antioxidant, anti-inflammatory and anti-fibrotic properties, should be designed and applied for the treatment and prevention of NAFLD and NASH in diabetic patients.

## Data Availability

The dataset used and/or analysed during the current study are available from the corresponding author on reasonable request.

## Abbreviations

T2DM: Type 2 diabetes mellitus
T1DM: Type 1 diabetes mellitus
ALA: Alpha-lipoic acid
DHLA: Dihydrolipoic acid
H_2_S: Hydrogen sulfide
CSE: Cystathionine γ-lyase
3-MST: 3-Mecaptopyruvate sulfurtransferase
PPG: DL-propargylglycine
NAFLD: Non-alcoholic fatty liver disease
NASH: Non-alcoholic steatohepatitis
ROS: Reactive oxygen species
GSH: Glutathione
SOD: Superoxide dismutase
IL-1β: Interleukin-1beta
IL-6: Interleukin-6
TNF-α: Tumor necrosis factor-alpha
TGF-β1: Transforming growth factor-beta1
α-SMA: Alpha-smooth muscle actin
MDA: Malondialdehyde
FFA: Free fatty acid
AMPK: Adenosine monophosphate-activated protein kinase
mTOR: Mammalian target of rapamycin
GLUT4: Glucose transporter protein subtype 4
K_ATP_: Adenosine triphosphate (ATP)-dependent potassium
LDL: Low-density lipoprotein
HDL: High-density lipoproteins
ALT: Alanine aminotransferase
AST: Aspartate aminotransferase
ALP: Alkaline phosphatase
FBG: Fasting blood glucose
HbA1c: Glycosylated haemoglobin
